# Why Do Doctors Not Advertise?

**Published:** 1893-05-13

**Authors:** 


The Hospital

An Institutional Journal of

Science, Medicine, Nursing, and Philanthropy.

*For Hospitals, Asylums, Infirmaries, Dispensaries, Medical Practitioners, Students, Nurses, and the Charitable Public.*

Vol. XIV.—No. 346.] May 13, 1893.

### Why do Doctors not Advertise?

Some persons have made fortunes by advertising, who, if they had not advertised, would have made no more than a bare living. Is there anything wicked in advertising, anything undignified? Certainly not! If a man has a good thing to sell, and can supply it in large quantities, why should he not let as many people as possible know of it? There is no reason whatever why he should not! Why, then, do doctors not avail themselves of this obvious method of improving their fortunes? This question is worth answering with some fulness at the present time.

There are two fundamental conceptions of the object of life which are different and opposite, and under one or other of these conceptions, as under a flag, all men range themselves, or are ranged by circumstances. The one conception is that a man's chief business is to push himself upward to the highest possible of the world's places at the greatest possible pace. The other is that a man's chief business is to do the particular work which falls to his lot as well as it can possibly be done. According to the former conception a man should constantly think of the end to be gained; according to the latter he should constantly think of the work to be done. We do not here argue whether of the two is the better or nobler conception of life. We merely affirm that there are these two leading conceptions; and that under the one or the other all men range themselves, doctors included.

Now certain persons, by natural instinct and voluntary choice, put their whole souls into their work without very much regarding the more or less remote destination to which the work leads. But there are others, and perhaps a far larger number, whose work itself is of such a kind that it compels steadfast and undivided attention to itself, to the more or less complete exclusion of the motive power of the fruits which it may ultimately yield to its cultivators. Such work, we insist, is the practice of medicine. The doctor, whether he will or not, is forced to belong to the class whose work, and not its rewards, constitutes the chief business. In saying this we are making no claim of superior merit for medical men. We are merely saying that their work is of such a nature, and of such commanding daily importance, that the average human being who gives himself to that work cannot withhold from it his full and entire energy and devotion from day to day. The necessity for giving to his work this absolute devotion gradually develops in the average medical man the conscious or unconscious conviction that the work itself and not its rewards is the true and proper object of life.

Here, then, at the very foundation of the doctor's activity, lies a fundamental reason why he should not advertise; and why, if he be honest, he will not. Pratically, every capable medical man can secure in a few years quite as many patients as he can attend to properly, and often a good many more. But when a doctor has secured as many patients as he can properly attend to the main object of his life is gained. He has as much work as he himself can personally do. He cannot multiply himself as the manufacturer, or the lawyer, or the shopkeeper can by means of clerks and assistants. His whole work is personal; he can do it, and he alone; and when his time is entirely filled up his capacity for dealing with more patients, that is for the extension of his business, is exhausted.

It may be contended that this is somewhat of a hardship for a practical, energetic, conquering mind: and so, indeed, it is. There are men in medicine who have no particular love for either money or fame, who yet chafe exceedingly at the painfully narrow limits within which their almost irrepressible energies are "cabined, cribb'd, and confined." These are the men who if they were soldiers would conquer continents, and if they were manufacturers would produce merchandise sufficient for the world. They find themselves out of sympathy with many of the ways of medicine; and their medical brethren, startled by their unusual energy, look upon them with suspicious antagonism, not knowing whether they may not be like "new wine in old bottles," and produce unpleasant catastrophes.

But loyalty of heart is what Carlyle has called a "thaumaturgic" quality, and compels the most restive of recruits to submit himself to discipline. The strongest spirits in medicine see, even more clearly than the weaker ones, that their art demands of them great self-repression, an infinity of patience and kindliness towards weak humanity, and an unwavering loyalty to the great and truly noble objects which all medicine has in view. For the sake of medicine, for the sake of man, of whom medicine is the true physical salvator, they will submit, if need be, to self-crucifixion, in order that there may be a resurrection to humane deeds and a worthy life.

Now this is a condition of things which makes advertising impossible for an honest medical man. To advertise would be to bring in more work than could possibly be well done. It would be as if a great artist, who could paint no more than twenty pictures a year, should suddenly announce his readiness to paint two or three hundred, or an indefinite number. Such an artist would promptly be ruined as an artist. His character and his capacity would be alike destroyed.

Is it, then, our contention that advertising doctors are and must be untrustworthy? It certainly is! And on this point we would do much to convince the public. For we think that if the public rightly appreciated the attitude of mind taken by the medical profession on the subject of advertising, it would be both sympathetic and grateful. After all, the world and our own country will only continue to stand and to prosper by the honest work it does. Let us picture to ourselves the whole of medical mankind leaving the ways of sober daily work, and proclaiming its merits, and ten times more than its merits, on the advertisers' hoardings. There would be a speedy collapse of everything? Whether would be better—that the few men who do advertise themselves and get rich thereby should give up advertising, or that the many should follow the example of the few and take to advertising? The question admits of only one answer.

At a period of much flux and change, and rushing hither and thither after novelty, some must stand for sobriety, for solid sense, and for plain, honest work. Happy are they who do so stand! On this noble rock modern medicine has firmly planted its feet. It is not obstinate; it is not averse from change. On the contrary, it is eager for progress and improvement. But it will not change without reason; it will not follow Will-o'-the-Wisps and self-seekers. Modern medicine aims only at the real, but hardly won, progress which will stand the tests of criticism and time.

It is sometimes complained of us that we are not enthusiastic—that we are so critical and cautious that the world loses patience with us. But this is not our fault. By nature medical men are just as enthusiastic, just as impulsive, and just as eager for rapid forward movement as men of every other class. But the nature of their work itself is such that calmness and sobriety, patience and watchfulness, become the ruling characteristics of the doctor, whether he will or not. Whatever class of men can live and thrive "up in the air," doctors cannot. Day by day they are forced down, and always down, upon the immovable bed-rock of reality. Of what use is it for them to tell a patient that he will live, when all the time it is but too obvious that he is dying? Most medical men would gladly conform themselves to the bustling and prosperous pace of the times if they could, but they cannot. Reality, slow reality, truth, unchangeable truth, are for ever their taskmasters; and whether good or ill fortune befall, the whips of those taskmasters must be obeyed. The truthful medical man cannot advertise and proclaim to the public capacities he does not possess, or achievements he has not achieved. Upon scientific medicine is laid the burden of plain, blunt honesty, even if comparative obscurity be the only reward of the enforced virtue.

